# Teaching of Nuclear Cardiology in Times of Pandemic: Transfer of a Case-based Interactive Course from Classroom to Distance Learning

**DOI:** 10.1055/a-1697-7795

**Published:** 2022-02-04

**Authors:** Laura Bell, Martin Lemos, Felix M. Mottaghy, Oliver Lindner, Alexander Heinzel

**Affiliations:** 1 74501 Child Neuropsychology Section, Department of Child and Adolescent Psychiatry, Psychosomatics and Psychotherapy, RWTH Aachen University Medical Faculty, Aachen, Germany; 2 74501 Audiovisual Media Center, RWTH Aachen University Medical Faculty, Aachen, Germany; 3 74501 Audiovisual Media Center, RWTH Aachen University Medical Faculty, Aachen, Germany; 4 568601 Department of Radiology and Nuclear Medicine, Maastricht University School for Oncology & Developmental Biology, Maastricht, Netherlands; 5 74501 Department of Nuclear Medicine, RWTH Aachen University Medical Faculty, Aachen, Germany; 6 39059 Institute of Radiology, Nuclearmedicine and Molecular Imaging, Heart and Diabetes Center North Rhine-Westphalia, Bad Oeynhausen, Germany; 7 74501 Department of Nuclear medicine, RWTH Aachen University Medical Faculty, Aachen, Germany; 8 196554 Department of Nuclear Medicine, Institute of Neurosciences and Medicine, Julich, Germany

**Keywords:** medizinische Lehre, Fernunterricht, Lernen auf Distanz, Nuklearkardiologie, Fallbasiertes Lernen, medical education, distance learning, nuclear cardiology, case-based learning

## Abstract

**Aim** While methods of independent study, such as problem-based learning, have been shown beneficial to students’ learning outcome and motivation to self-educate, these concepts are currently challenged by the pandemic. The aim of the current study was the evaluation of the transfer of an interactive nuclear cardiology teaching module to an online, distance learning setting *.* **Methods** Two-hundred-forty medical students completed and evaluated the teaching module in a classroom and 127 students in the distance learning setting. **Results** The interactive, problem-based teaching module was transferred well into the distance learning setting during the pandemic. However, while the presented results suggest that distance learning is a good substitute for classroom teaching when in-person teaching is not possible, the distance teaching module was perceived less efficient in its course didactics, demands as well as applicability than the same module in a classroom setting. **Conclusion** Although distance learning thus cannot entirely replace classroom education, it does provide a well-suited alternative method to teach particularly nuclear medicine and medicine in general. Future applications should offer introductory sessions, provide learning materials in advance and slow down the teaching pace to facilitate online, distance learning.

## Zusammenfassung

**Ziel** Während Methoden des Selbststudiums, wie z.B. das problembasierte Lernen, sich als vorteilhaft für den Lernerfolg und die Motivation von Studenten erwiesen, sich selbst weiterzubilden, werden diese Konzepte derzeit durch die Pandemie erschwert. Ziel der aktuellen Studie war die Evaluation des Transfers eines interaktiven nuklearkardiologischen Lehrmoduls in ein online-basiertes Fernstudium. **Methoden** Zweihundertvierzig Medizinstudierende absolvierten und bewerteten das interaktive Lehrmodul in einem Klassenzimmer und 127 Studierende in der Fernlernumgebung. **Ergebnisse** Das interaktive, problembasierte Lehrmodul konnte während der Pandemie gut in die Fernlernsituation übertragen werden. Obgleich die vorgestellten Ergebnisse darauf hindeuten, dass der Online-Unterricht ein guter Ersatz für den Präsenzunterricht ist, wenn dieser nicht möglich ist, wurde das Fernunterrichtsmodul in Bezug auf die Kursdidaktik, dessen Anforderungen sowie Anwendbarkeit als schlechter empfunden als das gleiche Modul in einer Präsenzveranstaltung. **Schlussfolgerung** Auch wenn das online-basierte Fernstudium den Unterricht im Klassenzimmer nicht vollständig ersetzen kann, stellt es eine gut geeignete alternative Methode dar, um Nuklearmedizin im Speziellen und Medizin im Allgemeinen zu lehren. Zukünftige Anwendungen sollten Einführungsveranstaltungen anbieten, Lernmaterialien im Voraus bereitstellen und das Unterrichtstempo verlangsamen, um Distanzkurs zu optimieren.

## Introduction

The teaching of nuclear medicine, especially within the frame of educating undergraduate medical students, is of utmost importance for the future development of the discipline. At best, it creates visibility, provides future physicians with the necessary knowledge to collaborate with nuclear medicine in interdisciplinary patient care, and it encourages candidates to choose a specialization in this field 1 . Just as the current pandemic situation poses a challenge for teaching of medicine in general, it has similarly affected the teaching of nuclear medicine 2 . During times of forced cancellation of many teaching activities, nuclear medicine has to be careful not to vanish from visibility in medical teaching. Therefore, concepts are needed that create sustainable learning experiences while respecting the rules of distance learning. One solution is the development of eLearning tools that make use of sophisticated viewing devices for nuclear medicine or hybrid imaging 3 4 5 . They offer great opportunities especially for teaching medical imaging. However, these tools take time to be build and implemented. Therefore, most often, they cannot be used as ad hoc solutions in times of unforeseen urgent need. In this paper, we aimed for a different approach. We tried to transfer a well-established class on nuclear cardiology from classroom teaching to distance learning using a video-conference tool. The challenge of this transfer was that the didactic concept of our class is inspired by student centered problem-based learning (PBL) focusing on interactions in small groups 6 . Several studies demonstrated the advantages of such approaches compared to traditional lecturer-centered learning. They lead to a higher student motivation, an increase in retrieval performance, and address more frequently higher-level competencies that refer not only to descriptive fact-based knowledge, but also to procedural knowledge as well as the training of soft skills 6 7 8 . Therefore, we intended to preserve the problem-based interactive character of the teaching module while applying distance learning. The aim of this study to evaluate the distance learning module of nuclear cardiology and was to compare the outcome of the evaluation with the evaluation of the same module in a classroom setting. This may reveal challenges and opportunities of distance learning as imposed by the current pandemic but may also indicate what role distance learning of nuclear medicine should and should not play in post-pandemic times.

## Materials and Methods

### Participants

The current study was approved by the local ethical committee (Medical Faculty, RWTH Aachen University). The volunteers were second-year students of the Medical Faculty of RWTH Aachen University that participated in either the teaching module in a classroom setting in 2020 or the same teaching module in a distance learning situation in 2021.

### Design of the nuclear cardiology course

The 60 minutes course is taught within the framework of the model degree program of medicine of the RWTH Aachen University (“Modellstudiengang Medizin”). It combines teaching of basic scientific preclinical and clinical knowledge throughout the curriculum to encourage horizontal and vertical integration of acquired knowledge (spiral curriculum 9 10 ). Thus, the course is linked to a preclinical course on cardiac physiology and a clinical lecture on nuclear cardiology. It is mandatory for all second-year medical students. The learning objectives are based on the German National Competence Based Catalogue of Learning Objectives for Undergraduate Medical Education (NKLM) ( http://www.nklm.de ), Chapter 15.5 and the related consensus paper of Marienhagenet al. 11 . The overall learning objective is to enable the students to understand and differentiate reversible and non-reversible myocardial ischemia diagnosed by myocardial perfusion SPECT. The study material consists of clinical case descriptions, myocardial perfusion SPECT images and related assignments. The overall program is designed as a case-based learning course in small groups. One class consists of 20 participants, that is subdivided into three smaller groups that are instructed to work on different clinical cases. In the classroom setting in 2020, all groups rotated and worked on all cases. In the distance learning situation in 2021, each group worked on one case only. After solving the case the original group/ all students will reunite, so that representatives of each small group can present their results to the other small groups. The didactic concept is closely related to classical PBL. Thus, the role of the lecturer is predominately to facilitate the interactive learning process, to model reasoning, and to encourage the students to develop and criticize their own clinical hypotheses 12 . However, due to time constrains, we do not follow the exact classical six steps of PBL. The course concept was originally developed for a classroom setting. In 2021, due to the coronavirus pandemic, we transferred this concept to a distance learning setting by using the video conference tool Zoom (Zoom Videos Communications, Inc. – San Jose, CA, USA). The division of the participants into small groups, and the respective group work was carried out with the help of the ‘breakout session’ function of Zoom. The lecturer split the participants into separate breakout rooms, while he moved between rooms, i.e., separate student groups, to stimulate the discourse between students, encouraging the learning process and to assist when needed. After solving the individual clinical cases, the breakout rooms were ended, and all groups came together again in the main room for their presentations in front of all students.

### Questionnaire

The authors developed a questionnaire to evaluate the teaching module in a classroom (2020) and a virtual, distance learning setting (2021). The questionnaire was originally developed for the evaluation of in-person teaching and consists of 22 items that can be categorized into six subscales, i.e., didactics (six items), demands on the students (three items), participation (two items), personal gain (five items), relevance to practical applicability (five items), and overall assessment (one item). Item 16, which evaluated the novel seminar type and its effect on learning success (personal gain subscale), was rated on a 5-point Likert-Scale ("1 – disagree", "2 – rather not agree", "3 – neither", "4 – rather agree", "5 – agree"). All remaining items were rated on a 7-point Likert-Scale ("1 – totally disagree", "2 – not agree", "3 – rather not agree", "4 – neither", "5 – rather agree", "6 – do agree", "7 – totally agree"). For an overview and a translation of all items in English, please see the results section for an overview of all items. Two open-ended questions additionally inquired about aspects that were particularly appreciated by the students and aspects that could be improved. For the evaluation of the teaching module in the distance learning situation, four items were added to address the distance learning setting (i.e., the availability of appropriate technical equipment, the access of the teaching material, the lack of social interactions with peers, and the suitability as substitute of a seminar in a classroom setting; see the results section for the open-ended questions. Further, an open-ended question was added to assess which aspects students would like to keep for future applications of the teaching module in the classroom setting. Students’ replies to the open-ended questions were independently divided into response groups by two raters. Inconsistent classifications were discussed, and the division was collectively decided upon.

### Analyses

Statistical analyses were performed in *R* 13 . Due to the skewed distribution and a lacking homogeneity of variance for several items, the evaluation of both teaching module settings (item-wise comparison and comparison of summary scores of each subscale), was compared using Mann-Whitney U tests. The tests were performed with the *R* package *stats* . The *rcompanion* package was used to calculate the effect size *r* 14 and plots were created with the ggplot2 package 15 .

## Results

Two-hundred-forty medical students (168 females, 66 males, 6 NA) evaluated the teaching module in the classroom setting and 127 medical students (92 females, 34 males, 1 NA) in the distance learning setting ( Table 1 ).

**Table:** **Table 1** Overall ratings per gender and setting (classroom (2020) vs. virtual, distance learning (2021)).

Year		Female	Male	NA
**2020**	*M (SD)*	5.90 (1.23)	6.03 (1.19)	5.80 (1.30)
**2021**		5.67 (1.24)	5.82 (1.07)	7.00 (-)
*Abbreviations* : *M* – Mean; *SD* – standard deviation. Note: The answer options for the overall rating were based on the following 7-point Likert scale: "1 – totally disagree", "2 – not agree", "3 – rather not agree", "4 – neither", "5 – rather agree", "6 – do agree", "7 – totally agree".				

**Fig. 1:**
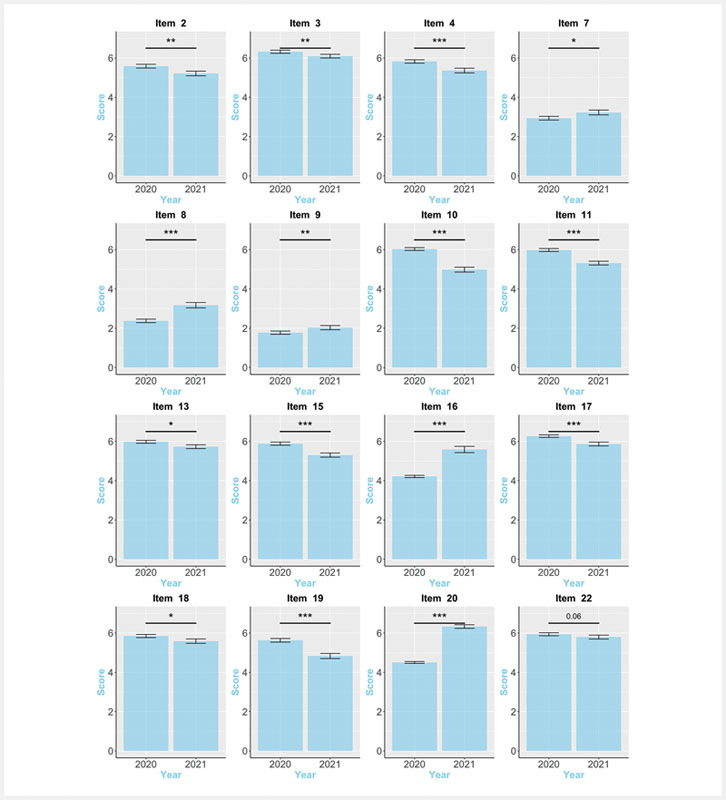
Differences in the evaluation of the teaching module between the classroom (2020) and the virtual, distance learning (2021) setting. *Note:* Only items are depicted, for which a statistically significant difference was found in the evaluation of the teaching module between settings (classroom (2020) vs. virtual, distance learning (2021)). The error bars represent the standard error. For the respective question of each item, see Tab. S1. The answer options for the 5-point Likert scale of item 16 were: "1- disagree", "2 – rather not agree", "3 – neither", "4 – rather agree", "5 – agree". The answer options for the remaining items were based on the following 7-point Likert scale: "1 – totally disagree", "2 – not agree", "3 – rather not agree", "4 – neither", "5 – rather agree", "6 – do agree", "7 – totally agree". *Significance* : * *p* < 0.05; ** *p* < 0.01; *** *p* < 0.001.

The overall rating of the teaching module was well across settings (classroom, distance learning; Table 2 ). Yet, the teaching module in classroom setting was evaluated marginally better than the teaching module in the distance learning setting, *U* = 15443, *p* = 0.06, *r* = 0.10 ( Table 2 ; Fig. 1 ; Item 22). This was similarly reflected in several items addressing course didactics, demands as well as applicability. That is, in the distance learning setting, it appears more difficult for the teacher to convey the information to the students in an interesting, vivid, and comprehensible manner (Item 2; 3; 4; Table 2 ). Additionally, the ability to critically reflect on the seminar’s topic (Item 19), to provide and critically discuss realistic examples (Item 18; 19) and to draw active links between theory and practice (Item 17) seems more difficult ( Table 2 ). Students experienced the seminar content as more difficult (Item 7), faster (Item 8) and time-consuming (Item 9) in the distance compared to the in-person teaching module ( Table 2 ). Furthermore, in the distance compared to the classroom module, group participation was perceived lower (Item 10; 11). Consequently, despite its positive evaluation, the online seminar was followed with less interest (Item 15) and was perceived as less helpful (Item 13) and marginally less relevant (Item 22) than the classroom module ( Table 2 ).

**Table:** **Table 2** Results per item and setting (classroom (2020) vs. virtual, distance learning (2021)).

Item	Subscale	Question	Points Likert-Scale	2020			2021				*U*	*p*		*r*
				*n*	*M*	*(SD)*		*n*	*M*	*(SD)*				
1	Didactics	I think the seminar gave a good overview of the subject area.	7	239	5.46	(1.47)		127	5.57	(1.10)	15461	0.76		0.02
2	Didactics	The lecturer often used examples that contributed to the understanding of the teaching content.	7	238	5.58	(1.47)		127	5.21	(1.33)	18100	0.001	**	0.17
3	Didactics	I think the lecturer responded appropriately to the students' questions and suggestions.	7	238	6.32	(1.25)		127	6.09	(1.07)	17955.50	0.001	**	0.17
4	Didactics	The lecturer has presented the topic in an interesting manner.	7	239	5.82	(1.32)		127	5.36	(1.34)	18653	< 0.001	***	0.20
5	Didactics	Throughout the seminar, I was always able to understand the structure of the seminar.	7	239	5.47	(1.54)		127	5.44	(1.37)	15788.50	0.51		0.03
6	Didactics	I think the lecturer used the available time well.	7	238	5.75	(1.51)		127	5.90	(1.12)	15273	0.86		0.01
7	Demands	The content of the seminar was too difficult for me.	7	239	2.94	(1.46)		127	3.23	(1.38)	13177.50	0.03	*	-0.11
8	Demands	The speed of material mediation/the seminar was too high for me.	7	239	2.38	(1.37)		127	3.17	(1.54)	10467	< 0.001	***	-0.26
9	Demands	The time required for the seminar was too much for me.	7	240	1.78	(1.27)		127	2.03	(1.20)	12634.50	0.003	**	-0.16
10	Participation	Most participants were actively involved.	7	240	6.03	(1.11)		126	4.98	(1.40)	22146.50	< 0.001	***	0.40
11	Participation	Most participants followed the seminar attentively and with interest.	7	240	5.98	(1.14)		126	5.32	(1.09)	20642.50	< 0.001	***	0.31
12	Personal Gain	The topics covered were significant and relevant to me.	7	238	5.78	(1.16)		126	5.58	(1.09)	16687	0.07		0.10
13	Personal Gain	I learned something useful and important in this seminar.	7	239	5.98	(1.15)		126	5.73	(1.08)	17404	0.01	*	0.13
14	Personal Gain	My understanding of the subject has developed through the seminar.	7	239	5.72	(1.25)		126	5.66	(1.08)	16013	0.30		0.05
15	Personal Gain	I was interested in the topic of the seminar.	7	239	5.88	(1.23)		126	5.30	(1.20)	19773	< 0.001	***	0.27
16	Personal Gain	I experienced the independent work on comprehension questions in the small group as motivating/ stimulating and it positively influenced my learning success.	5	238	4.21	(0.89)		126	5.59	(1.79)	5508	< 0.001	***	-0.53
17	Applicability	Links between theory and practice were demonstrated.	7	237	6.27	(1.02)		124	5.86	(1.09)	18463	< 0.001	***	0.22
18	Applicability	The lecturer illustrated the material with realistic examples.	7	238	5.85	(1.25)		124	5.59	(1.26)	16776.50	0.03	*	0.12
19	Applicability	He encouraged critical discussions about the topics.	7	236	5.63	(1.43)		124	4.83	(1.48)	19321.50	< 0.001	***	0.27
20	Applicability	I find the early discussion of clinical content motivating.	7	238	4.50	(0.73)		124	6.33	(1.03)	2222.50	< 0.001	***	-0.73
21	Applicability	The discussed topics were remote from life.	7	236	2.15	(1.66)		124	1.91	(1.00)	14182	0.61		-0.03
22	Overall Assessment	All in all, the attendance was of relevance.	7	225	5.94	(1.22)		124	5.80	(1.12)	15443	0.06		0.10
*Abbreviations: M* – Mean; *SD* – standard deviation; *U* – Mann-Whitney U test statistics; *p* – *p* -value; *r* – effect size. *Note:* All items were translated from German to English. The answer options for the 7-point Likert scale were: "1 – totally disagree", "2 – not agree", "3 – rather not agree", "4 – neither", "5 – rather agree", "6 – do agree", "7 – totally agree" and the answer options for the 5-point Likert scale were: "1- disagree", "2 – rather not agree", "3 – neither", "4 – rather agree", "5 – agree". *Significance* : * *p* < 0.05; ** *p* < 0.01; *** *p* < 0.001.														

Only the independent work in small groups (Item 16) and the early discussion of the seminar’s topic (Item 20) were rated better in the distance compared to the classroom module ( Table 2 ). For an overview of the differences between settings see Fig. 1 .

**Fig. 2:**
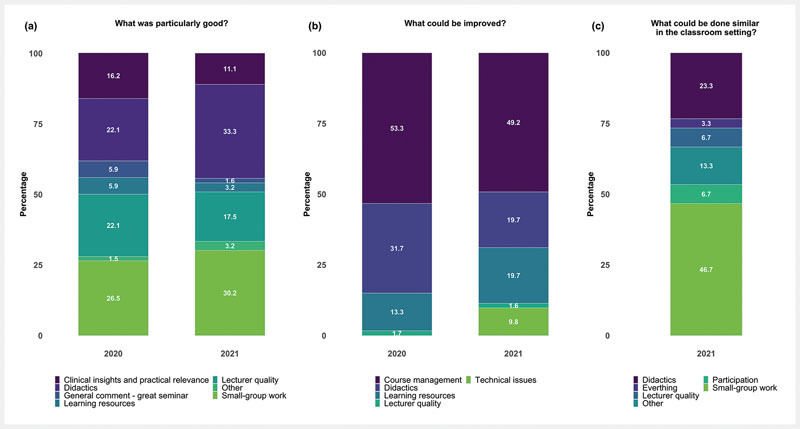
Replies to the Open-Ended Questions. **a** Responses to the open-ended question “What could be improved?” are depicted as percentages per response group and setting (classroom (2020) vs. virtual, distance learning (2021)). **b** Responses to the open-ended question “What was particularly good?” are depicted as percentages per response group and setting (classroom (2020) vs. virtual, distance learning (2021)). **c** Responses to the open-ended question “What could be done similar in the classroom setting” are depicted as percentages per response group for the virtual, distance learning teaching module in 2021.

Across both settings, the three most appreciated aspects of the teaching module mentioned in the open-ended questions were the small group work (classroom: 26.5%; distance learning: 30.2%), the didactics of the teaching module (classroom: 22.1%; distance learning: 33.3%), and the quality of the lecturer (classroom: 22.1%; distance learning: 17.5%). Next to the general appreciation of the interactive group work and the possibility to ask questions and receive valuable assistance and feedback by the lecturer, students’ positive, didactics-related comments were mainly related to the direct and active application of acquired knowledge. The three most frequently mentioned aspects for improvements were the course management (classroom: 53.3%; distance learning: 49.2%), the didactics (classroom: 31.7%; distance learning: 19.7%) and the learning resources (classroom: 13.3%; distance learning: 19.7%) across both settings. Course management related comments largely concerned the time allocation to the completion of the group work as well as the course integration into the curriculum. For the classroom setting, course management related answers were also addressing the order of the task completion (i.e., subsequent tasks were easier if task 1 was completed first). The didactics-related comments primarily raised suggestions for an initial introduction to the course structure and topic. In addition, students suggested that it would be beneficial to have learning resources available for preparation prior to the start and a summary after the teaching module. The students evaluating the teaching module in the distance learning setting suggested that the small group work and especially the course didactics should be maintained in a classroom-based format as soon as in-person meetings are possible again. For a comparison of all open-ended questions see Fig. 2 .

**Fig. 3:**
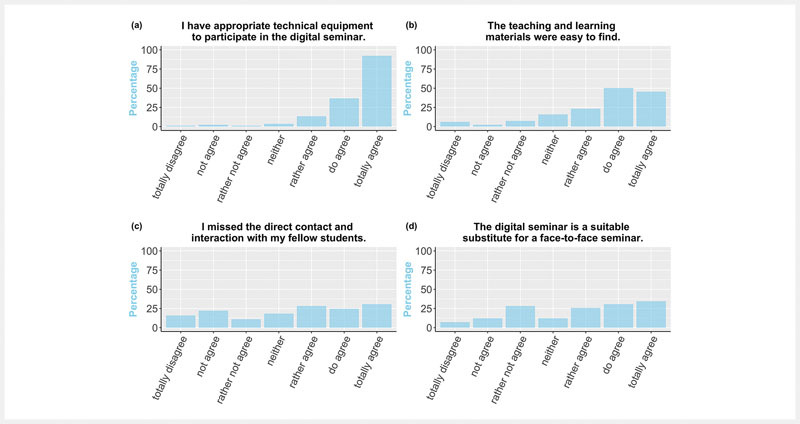
Answers to the items addressing the distance learning situation in 2021.

While most students reported having the right equipment and finding the learning materials easily for the distance learning module, responses to lack of direct contact and suitability as a substitute for a classroom module were divergent, see Fig. 3 .

## Discussion

Overall, the case-based interactive teaching module in nuclear cardiology was rated very good across settings. This is in line with prior evaluations of PBL-based learning in medical education 6 16 17 . In the current evaluation, particularly the interactive character with the self-employed identification of learning content and objectives, as well as the subsequent application of the acquired knowledge to different clinical cases, were appreciated by the students. Within these concepts, the role of the lecturer changes to that of a tutor who supports the learning process instead of teaching frontally. The positive results of both courses confirm the finding of Mayoet al. 18 and Steinert 19 . Accordingly, the role of the tutor as a metacognitive guide who encourages students to thinking critically about clinical cases is essential. Interestingly, the ratings on the appreciation of the independent work in small groups was higher in the distance learning than the classroom setting. This could be, *inter alia* , due to a general appreciation of modern, alternative curricular methods as well as a higher appreciation of the interactive character in the current situation. Given the current coronavirus pandemic and the advised social distancing, students may have particularly valued contact and interactive work with their fellow students. Indeed, recent studies on the health of students under lockdown indicated that students’ increased stress and worries during the pandemic, as well as the lack of social interaction and emotional support are associated with negative mental health trajectories 20 21 . Transferring the case-based interactive course from a classroom to a distance learning setting might have thus offered the students an alternative social interaction with their fellow students. In addition, unlike in the classroom setting, students in the distance learning setting worked only on one clinical case, which they subsequently presented to their peers. Students might have additionally appreciated the presentations and thus the immediate application of the acquired knowledge, which was indeed mentioned by the students within the open-ended questions on positive feedback. Nevertheless, students’ ratings on the suitability of the distance learning module as an appropriate substitute for the same teaching module in a classroom setting, as well as ratings on the absence of direct social contact, were divergent. One potential factor underpinning this diversity could be students’ personality traits (particularly agreeability and conscientiousness), which in turn not only leads to different preferences, but also to different coping mechanisms during a pandemic (e.g., Asselmannet al. 22 ).

Knowledge transfer and active engagement of students in an online module appears generally more difficult. While students appreciate the alternative teaching module and seem well equipped to take part in online modules, they perceived the online seminar as more difficult and time consuming than the same module in a classroom setting. Next to a lower teaching speed, online seminars might thus require alternative methods to increase the students’ interest as well as active participation. Face-to-face teaching primarily offers course didactic advantages, such as an easier opportunity to reinforce the active discussion of realistic examples and to draw comprehensible connections between theory and practice. The current findings are thereby consistent with a recent thematic review addressing the advantages and disadvantages of remote teaching in higher education during the pandemic across several disciplines 23 . While the overall transfer of the nuclear medicine course to a distance learning format was generally successfully and future, partial transfer of some modules to an online format may proof beneficial, the presented findings thus suggest that online seminars cannot entirely replace face-to-face instruction in classroom-based modules.

It should be noted that this is a first attempt to transfer the case-based interactive nuclear cardiology course from the classroom to a distance learning setting. Additional course management and technical issues, as reflected in the students’ replies to the open-ended questions, could thus have potentially contributed to the lower scores in distance learning setting. Future applications of a nuclear cardiology as well as other medical courses in a distance learning setting might consider an additional introduction to the course concept and the provision of study material in advance to prevent organizational issues, save time and to clarify potential questions and concerns that might hamper student participation. Challenges encountered during the preparation and delivery of distance learning are often of a different nature than challenges faced in classroom teaching. That is, for example, while the preparation of distance learning does not per se involve additional organizational effort in terms of room and time organization, an important prerequisite for distance learning is the organization of adequate technical equipment and often the replanning or didactic reorganization of face-to-face teaching 24 . With the growing number of online courses, whose development was additionally driven by the pandemic according to the thematic analysis by Erlichet al. 25 , a training of teachers in media didactics and qualitative adaptation of teaching material to distance learning is needed to optimize online education for both teachers and students. For a literature review on the effectiveness of distance learning and other aspects that should be considered when adapting classroom to online education, see Singh and Hurley 24 .

The presented findings should be evaluated with consideration of some limitations. First, the current evaluation only addressed aspects of students’ experience, but did not directly assess the learning outcome. Although an examination took place, nuclear medicine was only one of several topics within the exam, with only a small proportion of examination questions (approx. 7%). Therefore, the relation of both, student ratings and teaching outcome was not addressed within the current study und remains unclear. Studies indicate that student ratings of teaching effectiveness are positively related to student achievement 26 27 . However, others emphasize that student ratings may be biased by factors unrelated to teaching effectiveness, such as the difficulty of exams or the anticipation of getting a good grade 28 . Since our evaluation took place more than a month before the exam, it might be speculated that these factors were only of minor relevance to our evaluation. Ultimately, randomized controlled studies are needed that directly compare the learning outcome of distance learning methods with that of classroom-based methods outside a pandemic situation. Moreover, it should be noted that the emergence of the pandemic created unprecedented changes in social life and social interactions with various implications. Therefore, the comparability of the pre-pandemic and the pandemic teaching modules and student groups as well as the generalizability to distance learning settings independent of a pandemic might be limited. Further, albeit the current study did not use a previously validated questionnaire, it is not always easy or possible to develop questionnaires that can be applied to the often very individual, broader diversity of teaching. However, future evaluations should consider developing a questionnaire that would be applicable to nuclear medicine teaching modules in general. Finally, it should be noted that the current findings refer to a one-day course only and therefore might not be generalizable to longer course modules.

## Conclusion

Our results demonstrate that interactive teaching concepts are feasible and highly appreciated by the students during the current pandemic. However, while distance learning concepts can help to maintain visibility of the discipline and to create sustainable learning experiences, and a partial shift of teaching modules to online modules is certainly helpful, they cannot fully replace but potentially enhance face-to-face instruction in classroom-based modules.
